# Urinary Analysis*Read before the Medical Association of Georgia, April, 1895.

**Published:** 1896-03

**Authors:** Louis H. Jones

**Affiliations:** Atlanta, Ga., Professor Chemistry and Medical Jurisprudence, Atlanta Medical College; 206 Equitable Building


### Southern Medical Record.

A MONTHLY JOURNAL OF MEDICINE AND SURGERY.

VOL. XXVI. ATLANTA, GA., MARCH, 1896. No. 3.

### Original Articles.

### URINARY ANALYSIS.*

BY LOUIS H. JONES, A. M., M. D., ATLANTA, GA.,

Professor Chemistry and Medical Jurisprudence, Atlanta Medical College.

There is no secretion or excretion, no tangible state or condition of the body which so accurately shows the daily variations in waste and nutrition as the urine. Accepting this fact, the importance of frequent examinations of the urine can scarcely be overestimated, and we must class its careful study in clinical medicine as an absolute essential to correct diagnosis and rational therapeutics.

Fortunately, the rapid strides made in physiological chemistry, and the more general use of the microscope, afford us means for determining the composition of the urine with great accuracy and a minimum amount of labor.

No greater mistake can be made than to assume that the condition of the urine is an index to the state of the kidneys alone. Disorders of the brain, the liver, the heart, the stomach, alike tell their tale in altered conditions of the urine. Indeed, it would not be going too far to say that no considerable departure from the normal could exist for any length of time in the human organism without such change being recorded by either a pathological condition of the urine or a decided variation from its normal physiological state.

*Read before the Medical Association of Georgia, April, 1895.

It is, however, my intention to discuss only the most reliable and simple methods of detecting such changes, and to point out a few of the sources of error present in some of the methods in common use.

There are in making an examination of the urine four points *usually* determined, viz.: reaction, specific gravity, and the presence or absence of albumin and sugar. To these should always be added a knowledge of the amount passed in twenty-four hours, and the quantity of urea present, and where there is reason to suspect disease, or where the chemical analysis indicates it, the microscope should always complete the work.

The *reaction* can be readily determined by means of litmus paper, the only point worthy of consideration being, in the event of an alkaline reaction, to know whether this represents the state of the blood, or whether it is due to fermentative changes, by means of which the urea has been converted into ammonium carbonate. Should this be the cause of an alkaline reaction, the litmus paper will regain its red color upon drying and warming. This knowledge is valuable for the reason that the presence of a *volatile* alkali in freshly voided urine is almost invariably associated with a diseased condition of the bladder.

Grave pathological conditions are often indicated by considerable variation from the normal of specific gravity. The urinometers in common use I have found to be exceedingly unreliable, different instruments giving, with the same specimen, variations ranging from two to six points. This fact, together with the error arising from variations in temperature, and the fact that the specimen examined may not represent an average of the twenty-four hours, makes the specific gravity as often obtained of but little diagnostic value.

The chemical tests generally made of the urine are a reproach alike to the chemist and the physician. What is to be desired are tests which are reliable under all ordinary conditions, which can be made without the consumption of too much time, and which require a minimum amount of skill for their proper performance. It behooves the profession to find such methods, and having found them to adopt them to the exclusion of all less reliable and uncertain means. It shall be my aim not to discuss the many useless and tedious processes advocated from time to time, but to give the results of my experience with a view to eliciting from those present such criticism and additional information as they may deem profitable.

For the detection of albumin no less than twenty or thirty distinct tests have from time to time been warmly advocated. For many of these extreme delicacy has been claimed; for some justly so, but frequently at the expense of reliable and trustworthy results. What is to be desired is not increased delicacy, but greater certainty in our methods. It is generally conceded that the presence of a minute quantity of albumin in the urine is of but little diagnostic value, and that upon its existence *alone* renal disease cannot be predicated. The oldest and by far the most commonly used test is the heat and nitric acid test, and yet it is subject to certain sources of error, and among its advocates there is much disagreement as to the best method of applying it. At best, it is liable to give rise to two errors: first, in highly concentrated urines we will often obtain a precipitate of urates upon the addition of either nitric or acetic acids. This is due to the fact that the normal urates present in the urine are converted by the acid into acid urates, which, being more or less insoluble, are precipitated out as a diffuse cloudiness. Take any urine with a specific gravity of 1030, or more, and the acid may give this result. In the next place, mucin is precipitated by nitric acid, and in all cases where there is any considerable amount of mucus, the acid will yield results that are unreliable. Again, if the quantity of albumin be small, and only a few drops of acid added, the albumin may be converted into acid albumin, and no precipitate be obtained. We know that albuminous urine has, as a rule, a low specific gravity, and yet we often see reports of cases where the specific gravity is high and there is reported a trace of albumin. Now, in a highly concentrated urine the mucin is necessarily present in an appreciable quantity, and would, with either nitric or acetic acid, give results which might readily be mistaken for a slight albumin reaction. This can be avoided by first adding to the urine to be tested a solution of sodium chloride or of potassium ferrocyanide, either of which, if mixed with the urine *prior* to the addition of the acid, will prevent precipitation of mucin or urates. Personally, I am partial to the ferrocyanide, and have adopted this test for routine work in the office. It has to commend it both simplicity and reliability. It is made by adding to, say, two drachms of urine about a drachm and a half of a solution of potassium ferrocyanide (strength 1 to 20), and then adding ten or fifteen drops of acetic acid, when, if albumin be present, a distinct cloudiness will appear throughout the mixture.

Heller's test with cold nitric acid is preferable to the heat and nitric acid test, but possesses substantially the same objections, though in a less degree.

Where minute traces of albumin are suspected, or where doubt exists after the use of a test, the potassio-mercuric iodide may be used. This test, though subject to error, is exceedingly delicate, and may act as a check in doubtful cases, for where it gives a negative result we may feel sure of the absence of even a minute quantity of albumin. However, so little diagnostic value attaches to slight traces of albumin that extreme delicacy is rarely useful. The absolute amount of albumin present is, from a clinical and diagnostic standpoint, of minor importance, and we believe in a given case that the comparative amounts present from day to day, or week to week, is in the vast majority of cases all that will be required.

I especially desire to call attention to the fact that often much unnecessary confusion is brought about by speaking of the percentage of albumin found in a given specimen of urine. As a matter of fact, the actual *weight percentage* of albumin present in urine never exceeds three to four per cent., and rarely exceeds one per cent., and yet it is common to hear of urines containing from fifty to seventy-five per cent. of albumin, reference being had to the bulk of the precipitate formed, usually with heat and nitric acid. This must necessarily depend much upon the time allowed for settling, and is wholly inaccurate and unscientific.

The methods of detecting sugar in the urine are numerous. Quite a number are based upon the fact that grape sugar in a strongly alkaline solution will, especially when boiled, reduce cupric oxide to cuprous or suboxide; this is the principle involved in Trommer's test and in the use of Fehling's and

Haine's solutions. All things considered, these tests furnish us the best working test available. They are open to the objection that other substances sometimes present in urine will exert a like effect upon the copper salts, and this is especially true in tests requiring the use of a large amount of urine, as in Trommer's test. The use of Fehling's solution has the great disadvantage, that, owing to the presence in it of Rochelle salts, it is unstable, and unless freshly prepared is altogether unreliable. Haine's solution, the formula of which is, thirty grains of pure sulphate of copper dissolved in half an ounce of distilled water, then mixed with half an ounce of pure glycerine and five ounces of liquor potassa added, is stable and keeps indefinitely without change. About one drachm of this solution in a test-tube should be brought to a boil and five to ten drops (never more) of urine added; it should then be again boiled for half a minute when, if sugar be present, a precipitate of the red suboxide of copper is formed. The small quantity of urine used in this test practically does away with the reduction of the copper salts by any other substance than the grape sugar, and, if properly conducted, affords a simple and reliable test.

Boettger's test with bismuth is favored by some, but the salt is blackened by other substances than sugar, notably sulphur, and for this reason, if albumin be present, it must first be removed before adding the bismuth. Moreover, in all highly colored urines, the bismuth is more or less darkened and may give rise to doubt as to the presence of small amounts of sugar. The fermentation test sometimes used, requires too much time and is, moreover, entirely useless when sugar is present in small amounts, inasmuch as the urine will dissolve more or less carbon-dioxide and yield negative results. For detecting small quantities of sugar, the phenyl-hydrazin test is delicate and reliable, and its use applicable in every variety of urine. It requires time and considerable care for its performance, and is not suited to the needs of the busy practitioner.

The determination of urea is a matter much neglected, and yet a knowledge of the amount excreted is oftentimes of much value, and especially so from a prognostic standpoint. In cases of chronic interstitial nephritis, the daily loss of urea is a reliable indication of the manner in which the kidneys are performing their function, and a decided lessening of the amount may enable us to predict uræmic symptoms, which would otherwise have been a surprise. With the aid of the little instrument of Dr. Doremus, the urea may be quickly determined with sufficient accuracy for all practical purposes.

Such other pathological substances present in urine, as pus, blood, etc., can be most quickly and surely detected by means of the microscope.

Before closing I desire to call attention to the centrifugal method of obtaining urinary sediment for microscopic examination. By means of a small electric motor (designed by Dr. C. W. Purdy), carrying two arms with tubes attached, and which can be run by a small battery, the urinary sediment may be obtained in a few minutes, thus avoiding the twelve to twenty-fours delay occasioned by waiting for the sediment to collect in the bottom of the tube, and possesses the additional advantage of yielding a sediment free from and unaffected by the results of fermentation.

*206 Equitable Building.*

---

SHOULD DOCTORS WEAR BEARDS?—Dr. F. A. Colby, of Berlin, N. H., in a letter to the *Boston Medical and Surgical Journal*, discusses the danger of the practice. He cites a number of cases in which doctors by wearing beards, have conveyed the infection of diphtheria, etc. The responsibility of the surgeon in this matter is particularly insisted upon. Some time ago the *Medical Record* discussed this subject, advising not necessarily a total abolition of the beard, but restricting it to modest and sanitary limits. At that time, however, we received such severe criticisms from correspondents who had for years worn long and breezy whiskers, that it seemed wise that the subject be dropped. We shall follow with interest the progress of Dr. Colby's propaganda.—*Medical Record.*

